# Association between claims‐based setting of diagnosis and treatment initiation among Medicare patients with hepatitis C

**DOI:** 10.1111/1475-6773.14330

**Published:** 2024-05-21

**Authors:** Hao Zhang, Yuhua Bao, Kayla Hutchings, Martin F. Shapiro, Shashi N. Kapadia

**Affiliations:** ^1^ Department of Health Policy and Organization University of Alabama at Birmingham Birmingham Alabama USA; ^2^ Department of Population Health Sciences Weill Cornell Medicine New York City New York USA; ^3^ Department of Psychiatry Weill Cornell Medicine New York City New York USA; ^4^ Division of General Internal Medicine Weill Cornell Medicine New York City New York USA; ^5^ Division of Infectious Diseases Weill Cornell Medicine New York City New York USA

**Keywords:** claims‐based algorithm, linkage to care, setting of diagnosis

## Abstract

**Objective:**

To develop a claims‐based algorithm to determine the setting of a disease diagnosis.

**Data Sources and Study Setting:**

Medicare enrollment and claims data from 2014 to 2019.

**Study Design:**

We developed a claims‐based algorithm using facility indicators, revenue center codes, and place of service codes to identify settings where HCV diagnosis first appeared. When the first appearance was in a laboratory, we attempted to associate HCV diagnoses with subsequent clinical visits. Face validity was assessed by examining association of claims‐based diagnostic settings with treatment initiation.

**Data Collection/Extraction Methods:**

Patients newly diagnosed with HCV and continuously enrolled in traditional Medicare Parts A, B, and D (12 months before and 6 months after index diagnosis) were included.

**Principal Findings:**

Among 104,454 patients aged 18–64 and 66,726 aged ≥65, 70.1% and 69%, respectively, were diagnosed in outpatient settings, and 20.2% and 22.7%, respectively in laboratory or unknown settings. Logistic regression revealed significantly lower odds of treatment initiation after diagnosis in emergency departments/urgent cares, hospitals, laboratories, or unclassified settings, than in outpatient visits.

**Conclusions:**

The algorithm identified the setting of HCV diagnosis in most cases, and found significant associations with treatment initiation, suggesting an approach that can be adapted for future claims‐based studies.

**What is known on this topic**

- The setting where a medical condition is diagnosed can significantly influence subsequent healthcare, especially for conditions requiring coordination across different settings.
- The existing evidence is based on data from a single organization, thereby restricting its applicability to a broader context.
- No previous study has investigated methodologies for identifying the diagnosis setting from claims data.

**What this study adds**

- This study developed an algorithm which uses claims data to assign cases to clinical settings in which they were diagnosed.
- We utilized a claims‐based sample of Medicare patients newly diagnosed with hepatitis C virus (HCV), used the algorithm to assign cases to diagnostic sites, and assessed the association between the site of diagnosis and initiation of treatment.

## 1 INTRODUCTION

The setting where a medical condition is diagnosed can be a crucial factor affecting subsequent healthcare, particularly for conditions that require linkage to specialty providers in other settings.^1^, ^2^, ^3^, ^4^ Studies have found that different settings of diagnosis for human immunodeficiency virus (HIV) or hepatitis C virus (HCV) can be associated with varying rates of linkage to specialist care, with outpatient clinics showing higher linkage rates compared to inpatient settings or emergency departments (ED).^1^, ^2^, ^3^, ^4^ These studies have all relied on data from a single organization, limiting their generalizability. Research using population data is needed to confirm and quantify this association and to inform development of policies and guidelines aimed at improving linkage to care after diagnosis.

Healthcare claims data can be used to evaluate the relationship between the setting of diagnosis and linkage to care in broader populations. However, because claims data are generated for billing purposes, it is often unclear which claims are associated with the clinical encounter that led to a diagnosis. Although certain claims may contain coded indicators of the specific service setting, patients may have encounters billed by multiple entities, making it difficult to ascertain the correct setting of diagnosis. Furthermore, the existence of diverse coding schemes within claims data adds to the complexity of developing a standardized algorithm for determining setting of diagnosis. Consequently, there is currently no consensus on how to use claims data to identify the setting in which a specific disease was diagnosed.

To address this literature gap, we developed a claims‐based algorithm that systematically attempts to associate newly diagnosed cases with the settings where the diagnoses were made, using HCV as an example. HCV is an infectious disease that carries a significant public health burden. It is frequently associated with injection drug use and behavioral health problems, resulting in shame and stigmatization among affected individuals.^5^, ^6^ Stigmatization can deter patients with HCV from seeking treatment. Despite availability of highly effective direct‐acting antiviral (DAA) therapies since 2015, studies have found that relatively few patients receive treatment (estimates ranged from 5% to 30%).^7^, ^8^, ^9^, ^10^, ^11^ Studies in single organizations found that the rate of linkage to specialist care varied by site of testing (65%–78% in outpatient settings received linkage to care vs. 30%–57% in inpatient settings).^2^, ^4^

Our algorithm assigns HCV cases to diagnostic settings using a combination of place‐of‐service codes, revenue center codes, and other information available in claims data, and is transparently reported to support reproducibility in studies of other conditions. To assess the algorithm's face validity, we examined the association between the claims‐based setting of diagnosis and treatment initiation with DAAs in a sample of Medicare patients newly diagnosed with HCV.

## 2 METHODS

### 2.1 Study design and data source

This study used Medicare data from a 100% sample of Medicare enrollees with at least one International Classification of Diseases 9th revision (ICD‐9) or 10th revision (ICD‐10) diagnosis of HCV in fee‐for‐service (FFS) Medicare claims during 2014–2019. Files used by our algorithm include the Master Beneficiary Summary file, Medicare Provider Analysis and Review (MedPAR) file, Outpatient file, Carrier file, and Part D Event (PDE) file.^12^ The study was approved by the institutional review board of Weill Cornell Medicine.

### 2.2 Algorithm for setting of diagnosis determination

Our algorithm classifies diagnostic settings into seven broad categories: hospital, ED/urgent care, outpatient, post‐acute/subacute/long‐term care, laboratory, other unclassified settings, and unknown. Table 1 provides a detailed list of the healthcare settings included in each category.

**TABLE 1:** Categorization of the setting of diagnosis into seven broad categories.

Categories	Included settings
Hospital	Short‐stay hospitals, long‐stay hospitals, residential mental health facilities, and residential substance use facilities.
ED/urgent care	ED, urgent care clinics.
Outpatient	Offices, health clinics, ambulatory surgical centers, mobile units, nonresidential substance use treatment facilities, outpatient hospitals, and telehealth.
Post‐acute/subacute/long‐term care	Skilled nursing facilities, custodial care facilities, inpatient or outpatient rehabilitation facilities, and assisted living facilities.
Laboratory	Laboratory
Other unclassified	Ambulance, hospice, home, school, homeless shelters, and correctional facilities.
Unknown	Missing facility codes or codes do not imply setting types.

*Note*: This table shows the seven broad categories our algorithm used and the settings in each category. Our algorithm used a combination of place‐of‐service codes, revenue center codes, and other information available in claims data, and categorized the setting of diagnosis into one of the seven broad categories. ED, emergency department.

This algorithm employs different variables to determine the setting of diagnosis based on the type of files associated with the index diagnosis. In cases where the index diagnosis is identified from the MEDPAR file, the algorithm classifies the setting of diagnosis as either a hospital (for short‐term or long‐term stay hospitals) or post‐acute/subacute/long‐term care (for skilled nursing facilities or SNFs), utilizing the short stay/long stay/SNF indicator in the MEDPAR file. If the index diagnosis is identified through Outpatient or Carrier claims, the algorithm extracts the Revenue Center Code (in Outpatient claims) or Place of Service code (in Carrier claims) and employs crosswalks developed by the research team to establish links between these codes and the seven broad categories (Appendices S2 and S3).

When multiple records are associated with an HCV diagnostic code on the same day and indicate different settings (based on the rules described above), it is essential to assign a single diagnostic setting for each case. To achieve this, the algorithm employs a hierarchical rule to prioritize certain settings over others, based on an understanding of the dominant healthcare event that is likely driving the index diagnosis. The hierarchy of settings is: hospital > ED/urgent care > post‐acute/subacute/long‐term care > outpatient > other unclassified settings > unknown > laboratory. For instance, if claims from both a doctor's office and a laboratory are present on the date of the index HCV diagnosis, “outpatient” is designated as the setting of diagnosis.

**FIGURE 1:**
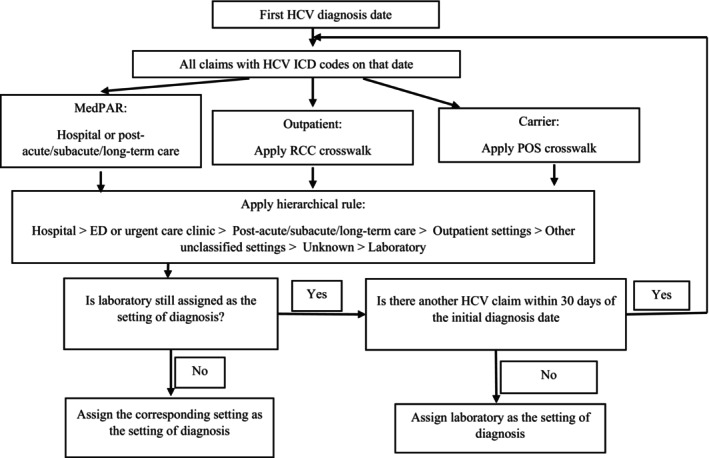
Algorithm to determine the setting of hepatitis C virus diagnosis using Medicare claims data. When laboratory was assigned as the initial setting diagnosis, this algorithm employs a loop which is repeated until a setting other than the laboratory is found or 30 days post the initial diagnosis is reached. The algorithm assigns a single setting to each patient. HCV, hepatitis C virus; ED, emergency department; MedPAR, Medicare provider analysis and review file; POS, place of service code; RCC, revenue center code.

The laboratory setting is given the lowest priority because it is uncommon for providers in a laboratory to make independent diagnoses. In cases where only laboratory claims are associated with an index HCV diagnosis, the next available HCV‐related claim within 30 days of the index diagnosis is used to assign the setting of diagnosis. This process is repeated if laboratory is still assigned to the next available claim and until a different setting was assigned or 30 days post the index diagnosis has been reached (Figure 1).

### 2.3 Applying the algorithm to hepatitis C virus sample

Our study sample included Medicare patients newly diagnosed with HCV between January 2015 and June 2019. Following existing literature, new HCV diagnoses were identified based on a Current Procedural Terminology code for an HCV ribonucleic acid (RNA) test followed by at least one chronic or acute HCV ICD diagnostic code within 180 days.^13^ (Appendix S1) We restricted our sample to those continuously enrolled in FFS Medicare Parts A, B, and Part D for 12 months before and 6 months after the month of the index HCV diagnosis. Among the patients who met the diagnosis and enrollment criteria, a small proportion (*n* = 9256) who filled DAA prescriptions before the index diagnosis were excluded from the final sample because we could not determine date of initial diagnosis.

We defined the date of new HCV diagnosis to be the first date when a patient met the diagnosis definition, that is the date of the ICD‐code. We identified all MedPAR, outpatient, and carrier claims with HCV ICD diagnostic codes on the index date, and applied the algorithm to determine the diagnostic setting.

### 2.4 Association between setting of diagnosis and direct‐acting antiviral initiation

To assess the face validity of the claims‐based assignment of cases to diagnostic settings, we tested the association of these assigned settings with DAA initiation. Patients were considered to have initiated DAA treatment if they had at least one filled DAA prescription within 180 days after the index diagnosis. DAA prescriptions were identified using National Drug Codes from the PDE file (Appendix S4). Multivariate logistic regressions were estimated adjusting for year of diagnosis, patient demographics including age, race/ethnicity, sex, Medicare‐Medicaid dual enrollment, and comorbidities. Because of the small sample sizes associated with some settings, we collapsed “post‐acute/subacute/long‐term care” and “other unclassified settings” (“other unclassified settings” hereafter), and “laboratory” and “unknown” settings (“laboratory or unknown settings” hereafter) in this step. Race‐ethnicity was based on the beneficiary race code recorded by the Social Security Administration and an algorithm developed by Research Triangle Institute which provided greater validity than the Medicare enrollment database race variable.^14^ Descriptive statistics were derived for demographic and comorbidity variables for patients diagnosed in each setting. Patients aged 18–64 and those 65 or older were analyzed separately considering their distinct healthcare utilization and spending patterns.^15^, ^16^, ^17^ For logistic regression results, we report predicted probabilities, which are calculated using the STATA “margins” command.

We further conducted two sensitivity analyses. First, 90‐ and 270‐day windows were used instead of the 180‐day window to capture DAA treatment initiation. Second, we employed a more stringent definition for HCV diagnosis, which required an RNA test followed by at least 2 ICD diagnoses on different service dates.

All analyses were performed using *SAS* 9.4.

## 3 RESULTS

### 3.1 Sample characteristics

Among the 104,454 patients aged 18–64 and the 66,726 patients aged 65 or older, most were male, non‐Hispanic White, and aged 55–74. Approximately 30% of patients initiated DAA treatment within 180 days of their index HCV diagnosis. Compared with patients 65 or older, patients aged 18–64 were more likely to be dually enrolled in Medicare and Medicaid and have opioid use disorder, alcohol use disorder, HIV, and mental health disorder. Details of the distribution of the demographics and comorbidities of the study sample are available in Appendix S7.

### 3.2 Determining the setting of diagnosis

Of the 104,454 patients aged 18–64, 55,999 had HCV claims from a single setting on the index date, and 48,455 from multiple settings. Among patients with HCV claims from a single setting, the laboratory setting was initially assigned to 34,706 patients. Of these, 15,002 had additional HCV claims within 30 days, and 13,653 were ultimately assigned a different diagnostic setting (Appendix S5). Of the 66,726 HCV patients in the ≥65 cohort, 38,665 had HCV claims from a single setting on the index date, and 28,061 from multiple settings. Among those with HCV claims from a single setting, laboratory was initially assigned to 24,139 patients. Of these, 10,049 had additional HCV claims within 30 days, and 9000 were ultimately assigned a different diagnostic setting (Appendix S6).

After collapsing groups with small sample size, the majority of patients were diagnosed in outpatient settings (70.1%, 18–64; 69.5%, 65 or older), followed by laboratory or unknown settings (20.2%, 18–64; 22.7%, 65 or older), hospitals (4.4%, 18–64; 3.2%, 65 or older), other unclassified settings (3.0%, 18–64; 3.3%, 65 or older), and ED/urgent care (2.3%, 18–64; 1.3, 65 or older). Patients diagnosed in hospitals or ED/urgent care were more likely to have opioid use disorder, alcohol use disorder, advanced liver disease, HIV, and mental health disorder, and less likely to be non‐Hispanic White. (Table 2).

**TABLE 2:** Demographic, comorbidity, and direct‐acting antiviral initiation characteristics of patients by claims‐based setting of diagnosis.

	ED or UC	Hospital	Outpatient	Other unclassified	Laboratory or unknown	*p*‐value^a^
a. Aged 18–64 at diagnosis						
*N*	2416	4603	73,226	3087	21,123	
Year of diagnosis						<0.001
2015	2.0%	3.6%	72.1%	2.7%	19.7%	
2016	2.3%	4.3%	69.6%	3.0%	20.9%	
2017	2.4%	4.7%	68.7%	3.0%	21.2%	
2018	2.9%	5.5%	68.7%	3.3%	19.7%	
2019	2.8%	5.7%	69.5%	3.2%	18.9%	
Sex						<0.001
Male	53.1%	59.3%	57.8%	58.5%	56.3%	
Female	46.9%	40.7%	42.2%	41.5%	43.7%	
Race						<0.001
White	63.7%	59.6%	66.6%	65.1%	66.6%	
Black	23.5%	26.2%	22.1%	24.2%	20.9%	
Hispanic	9.9%	10.2%	8.6%	8.1%	9.8%	
Asian/Pacific Islander	0.6%	0.9%	0.7%	1.1%	0.9%	
Other or unknown	2.4%	2.6%	1.1%	1.7%	1.8%	
Age						<0.001
> = 18 and <35	8.7%	9.1%	4.7%	2.6%	4.8%	
> = 35 and <55	43.5%	39.6%	38.1%	31.8%	38.1%	
> = 55 and <65	47.8%	51.4%	57.2%	65.7%	57.2%	
> = 65 and <75						
> = 75						
Medicare‐Medicaid dual enrollment						<0.001
Yes	86.3%	85.4%	81.0%	83.7%	80.4%	
No	13.7%	14.6%	19.0%	16.3%	19.6%	
Comorbidity						
Opioid use disorder	36.1%	39.8%	22.7%	20.6%	22.3%	<0.001
Alcohol use disorder	31.8%	36.8%	19.3%	22.3%	16.3%	<0.001
Advance liver disease	22.8%	25.5%	16.4%	28.1%	13.8%	<0.001
HIV	9.5%	10.0%	7.4%	7.1%	8.0%	<0.001
Mental health disorder	80.0%	80.0%	67.5%	76.3%	66.2%	<0.001
DAA initiation within 180 days	12.6%	8.1%	35.3%	21.1%	20.0%	<0.001
b. 65 or older at diagnosis						
*N*	833	2116	46,386	2221	15,170	
Year of diagnosis						<0.001
2015	0.9%	2.4%	72.5%	2.8%	21.4%	
2016	1.2%	3.1%	69.2%	3.2%	21.4%	
2017	1.3%	3.1%	68.5%	3.3%	23.9%	
2018	1.7%	4.0%	67.4%	3.8%	23.1%	
2019	1.4%	4.0%	68.7%	4.3%	21.6%	
Sex						<0.001
Male	58.8%	38.1%	56.1%	60.0%	53.5%	
Female	41.2%	61.9%	44.0%	40.0%	46.5%	
Race						<0.001
White	48.3%	49.6%	56.9%	55.1%	61.0%	
Black	37.0%	35.2%	27.4%	31.1%	21.8%	
Hispanic	9.6%	9.8%	8.7%	8.4%	8.6%	
Asian/Pacific Islander	2.4%	3.0%	4.4%	2.8%	6.0%	
Other or unknown	2.8%	2.5%	2.6%	2.6%	2.7%	
Age						<0.001
> = 18 and <35						
> = 35 and <55						
> = 55 and <65						
> = 65 and <75	84.2%	81.3%	82.5%	78.8%	81.2%	
> = 75	15.9%	18.7%	17.5%	21.2%	18.8%	
Dual eligibility						<0.001
Yes	68.4%	62.7%	50.5%	33.2%	54.6%	
No	31.6%	37.3%	49.5%	66.8%	45.4%	
Comorbidity						
Opioid use disorder	19.2%	17.8%	7.7%	10.1%	7.1%	<0.001
Alcohol use disorder	21.6%	20.6%	9.0%	15.2%	7.0%	<0.001
Advance liver disease	28.6%	32.0%	19.4%	28.1%	16.1%	<0.001
HIV	3.6%	6.1%	2.9%	5.1%	2.8%	<0.001
Mental health disorder	60.0%	62.6%	39.9%	64.5%	38.9%	<0.001
DAA initiation within 6 months	13.2%	8.5%	38.5%	18.7%	18.3%	<0.001

*Note*: This table shows the sample size, demographic covariates, and comorbidities for new HCV patients diagnosed in different settings. Separate tables were reported for patients aged 18–64 and 65 or older. Research Triangle Institute definition was used for the race‐ethnicity variable. *p*‐values were calculated using Chi‐squared test.

Abbreviations: DAA, direct‐acting antiviral; ED, emergency department; HIV, human immunodeficiency virus; UC, urgent care clinic.

^a^ *p*‐value for chi‐square test.

### 3.3 HCV treatment initiation by setting of diagnosis

Patients whose diagnosis was assigned to outpatient settings had the highest treatment initiation rate (35.3%, 18–64; 38.5%, 65 or older). Hospitals (8.1%, 18–64; 8.5%, 65 or older) and ED/urgent care (12.6%, 18–64; 13.2%, 65 or older) were associated with the lowest initiation rates. Difference in the predicted probability of treatment initiation from the logistic regression model ranged from −29.2% (95% CI: −30.5%, −27.8%) for hospitals to −14.6% (95% CI: −16.1%, −13.2%) for other unclassified settings, compared to outpatient settings (Appendix S8). Results from the sensitivity analyses were consistent with the findings from the main analyses. (Appendices S9–S12).

## 4 DISCUSSION

This study employed a claims‐based algorithm to associate newly diagnosed cases of HCV with diagnostic settings. We demonstrated a strong association between claims‐based setting of diagnosis and likelihood of HCV treatment initiation. Our algorithm holds potential for application in other disease contexts, particularly those associated with stigma or in populations in which follow‐up care for positive test results may be challenging.

This study is the first of which we are aware that systematically develops and reports on a claims‐based algorithm to determine the setting in which a diagnosis is made. One recent study examined the association between a claims‐based setting of diagnosis and treatment engagement among Medicaid patients with opioid use disorder,^18^ without providing detailed information on how diagnosis setting was determined. This highlights the need for a well‐defined and replicable approach. Our study responds to this need.

This study's findings highlighted the potential role of diagnostic setting in understanding and addressing disparities in treatment initiation and access to care in general, especially for stigmatized conditions that may require linkage to care in other settings. The finding of significantly lower DAA initiation rate for patients diagnosed in hospital or ED/urgent care settings was consistent with previous research.^2^, ^4^ These findings highlight the importance of considering sites of diagnosis when studying treatment uptake. This also underscores the need for healthcare system efforts to link patients to care after diagnosis in acute settings.

Even after reassignment of many patients using the algorithm, approximately 1/5 continued to be assigned to the laboratory as the setting of HCV diagnosis, raising questions about interpretation. Laboratories play a vital role in the diagnostic process, but typically do not provide an independent diagnosis. One explanation is that HCV diagnostic codes were used to bill for laboratory tests regardless of whether the tests yielded positive results. As a result, patients who did not actually have the condition may have been incorrectly classified as diagnosed patients in the study sample. Another possible explanation is that patients tested positive (and received a diagnosis from the laboratory) but did not follow up with the providers who ordered the test or with other potential treating providers.

Outside of HCV, this approach may be applied to evaluating service delivery for other conditions, especially those for which transitions of care between healthcare settings may be a barrier to quality or contribute to disparities. For instance, single‐institution studies have shown suboptimal rates of referral to HIV pre‐exposure prophylaxis for individuals diagnosed with sexually transmitted diseases in the emergency department,^19^ or for hospitalized individuals with new diagnoses of diabetes.^20^

In addition, our approach can provide insights into the frequency of diagnosis across healthcare settings. This information can help healthcare administrators and policymakers streamline resource allocation, enhance facility planning, and ensure efficient patient management across healthcare settings. Information on diagnostic settings could also enable evaluations of healthcare quality for some medical conditions.

### 4.1 Limitations

This study has several limitations. First, without access to data (e.g., electronic health records) on the actual setting in which a patient received their diagnosis, we were not able to directly assess the validity of the algorithm developed. The analysis assessing the association between claims‐based setting and treatment initiation rate supported face validity. Second, the hierarchical rule to assign an index diagnosis to a single setting was based on understanding about where the dominant healthcare event is likely to take place when multiple events in multiple settings co‐occur on the same day. Such a rule may not always align with the specific setting where the diagnosis was initially made. Third, like in all analyses using claims data, the new HCV diagnosis identified may not represent the very first diagnosis received by a given patient. The requirement of 12 months of continuous enrollment prior to the new HCV diagnosis mitigated the concern.

## 5 CONCLUSIONS

In summary, we developed an algorithmic approach to determining the setting of diagnosis using claims data, and demonstrated that the claims‐based setting of diagnosis was associated with treatment initiation in HCV. This approach provides a template for future claims‐based studies to operationalize a setting‐of‐diagnosis variable when examining healthcare utilization and access.

## FUNDING INFORMATION

This work was funded by grant R01 DK123205 from the National Institute of Diabetes, Digestive, and Kidney Diseases and grants K01 DA048172 and P30 DA040500 from the National Institute on Drug Abuse.

## Supporting information


**Appendix S1.** Supporting information.
