# Supplementary material for: Association between claims‐based setting of diagnosis and treatment initiation among Medicare patients with hepatitis C
Source: Health Serv Res. 2024 May 21;59(4):e14330. doi: 10.1111/1475-6773.14330 (PMC11249812; doi:10.1111/1475-6773.14330)
Supplement: Supplementary file 1 — Appendix S1. Supporting information. [file HESR-59-0-s001.docx]

**Association between claims-based setting of diagnosis and treatment initiation among Medicare patients with hepatitis C**

Appendix

Appendix S1. Codes for hepatitis C diagnosis.

|  | Coding system | Codes |
| --- | --- | --- |
| Chronic hepatitis C | ICD-9 | 070.44, 070.54, 070.70, 070.71, 070.51, V02.62 |
|  | ICD-10 | B18.2, Z22.52, B19.20, B19.21 |
| Acute hepatitis C | ICD-9 | 070.41 |
|  | ICD-10 | B17.10, B17.11 |
| RNA test | CPT | 87520, 87521, 87522 |

Abbreviations: ICD - International Classification of Diseases; RNA - ribonucleic acid; CPT - Current Procedural Terminology.

Appendix S2. Revenue center code to setting of diagnosis crosswalk.

| **RCC** | **Name** | **Broad Category** |
| --- | --- | --- |
| 0001 | Total charge | Exclude |
| 0022 | SNF claim paid under PPS submitted as type of bill (TOB) 21X. | Post Acute or LTC |
| 0023 | Home Health services paid under PPS submitted as TOB 32X and | Other unclassified |
| 0024 | Inpatient Rehabilitation Facility services paid under PPS | Post Acute or LTC |
| 0100 | All-inclusive rate - room and board plus ancillary | Inpatient |
| 0101 | All-inclusive rate - room and board | Inpatient |
| 0110 | Private medical or general-general classification | Inpatient |
| 0111 | Private medical or general-medical/surgical/GYN | Inpatient |
| 0112 | Private medical or general-OB | Inpatient |
| 0113 | Private medical or general-pediatric | Inpatient |
| 0114 | Private medical or general-psychiatric | Inpatient |
| 0115 | Private medical or general-hospice | Other unclassified |
| 0116 | Private medical or general-detoxification | Inpatient |
| 0117 | Private medical or general-oncology | Inpatient |
| 0118 | Private medical or general-rehabilitation | Post Acute or LTC |
| 0119 | Private medical or general-other | Inpatient |
| 0120 | Semi-private 2 bed (medical or general) general classification | Inpatient |
| 0121 | Semi-private 2 bed (medical or general) medical/surgical/GYN | Inpatient |
| 0122 | Semi-private 2 bed (medical or general)-OB | Inpatient |
| 0123 | Semi-private 2 bed (medical or general)-pediatric | Inpatient |
| 0124 | Semi-private 2 bed (medical or general)-psychiatric | Inpatient |
| 0125 | Semi-private 2 bed (medical or general)-hospice | Other unclassified |
| 0126 | Semi-private 2 bed (medical or general)-detoxification | Inpatient |
| 0127 | Semi-private 2 bed (medical or general)-oncology | Inpatient |
| 0128 | Semi-private 2 bed (medical or general)-rehabilitation | Post Acute or LTC |
| 0129 | Semi-private 2 bed (medical or general)-other | Inpatient |
| 0130 | Semi-private 3 and 4 beds-general classification | Inpatient |
| 0131 | Semi-private 3 and 4 beds-medical/surgical/GYN | Inpatient |
| 0132 | Semi-private 3 and 4 beds-OB | Inpatient |
| 0133 | Semi-private 3 and 4 beds-pediatric | Inpatient |
| 0134 | Semi-private 3 and 4 beds-psychiatric | Inpatient |
| 0135 | Semi-private 3 and 4 beds-hospice | Other unclassified |
| 0136 | Semi-private 3 and 4 beds-detoxification | Inpatient |
| 0137 | Semi-private 3 and 4 beds-oncology | Inpatient |
| 0138 | Semi-private 3 and 4 beds-rehabilitation | Post Acute or LTC |
| 0139 | Semi-private 3 and 4 beds-other | Inpatient |
| 0140 | Private (deluxe)-general classification | Inpatient |
| 0141 | Private (deluxe)-medical/surgical/GYN | Inpatient |
| 0142 | Private (deluxe)-OB | Inpatient |
| 0143 | Private (deluxe)-pediatric | Inpatient |
| 0144 | Private (deluxe)-psychiatric | Inpatient |
| 0145 | Private (deluxe)-hospice | Other unclassified |
| 0146 | Private (deluxe)-detoxification | Inpatient |
| 0147 | Private (deluxe)-oncology | Inpatient |
| 0148 | Private (deluxe)-rehabilitation | Post Acute or LTC |
| 0149 | Private (deluxe)-other | Inpatient |
| 0150 | Room & Board ward (medical or general)-general classification | Inpatient |
| 0151 | Room & Board ward (medical or general)-medical/surgical/GYN | Inpatient |
| 0152 | Room & Board ward (medical or general)-OB | Inpatient |
| 0153 | Room & Board ward (medical or general)-pediatric | Inpatient |
| 0154 | Room & Board ward (medical or general)-psychiatric | Inpatient |
| 0155 | Room & Board ward (medical or general)-hospice | Other unclassified |
| 0156 | Room & Board ward (medical or general)-detoxification | Inpatient |
| 0157 | Room & Board ward (medical or general)-oncology | Inpatient |
| 0158 | Room & Board ward (medical or general)-rehabilitation | Post Acute or LTC |
| 0159 | Room & Board ward (medical or general)-other | Inpatient |
| 0160 | Other unclassified Room & Board-general classification | Inpatient |
| 0164 | Other unclassified Room & Board-sterile environment | Inpatient |
| 0167 | Other unclassified Room & Board-self care | Inpatient |
| 0169 | Other unclassified Room & Board-other | Inpatient |
| 0170 | Nursery-general classification | Inpatient |
| 0171 | Nursery-newborn level I (routine) | Inpatient |
| 0172 | Nursery-premature newborn-level II (continuing care) | Inpatient |
| 0173 | Nursery-newborn-level III (intermediate care) | Inpatient |
| 0174 | Nursery-newborn-level IV (intensive care) | Inpatient |
| 0179 | Nursery-other | Inpatient |
| 0180 | Leave of absence-general classification | Unknown |
| 0182 | Leave of absence-patient convenience charges billable | Unknown |
| 0183 | Leave of absence-therapeutic leave | Unknown |
| 0184 | Leave of absence-ICF mentally retarded-any reason | Unknown |
| 0185 | Leave of absence-nursing home (hospitalization) | Unknown |
| 0189 | Leave of absence-other leave of absence | Unknown |
| 0190 | Subacute care - general classification | Post Acute or LTC |
| 0191 | Subacute care - level I | Post Acute or LTC |
| 0192 | Subacute care - level II | Post Acute or LTC |
| 0193 | Subacute care - level III | Post Acute or LTC |
| 0194 | Subacute care - level IV | Post Acute or LTC |
| 0199 | Subacute care - other | Post Acute or LTC |
| 0200 | Intensive care-general classification | Inpatient |
| 0201 | Intensive care-surgical | Inpatient |
| 0202 | Intensive care-medical | Inpatient |
| 0203 | Intensive care-pediatric | Inpatient |
| 0204 | Intensive care-psychiatric | Inpatient |
| 0206 | Intensive care-post ICU; redefined as intermediate ICU | Inpatient |
| 0207 | Intensive care-burn care | Inpatient |
| 0208 | Intensive care-trauma | Inpatient |
| 0209 | Intensive care-other intensive care | Inpatient |
| 0210 | Coronary care-general classification | Inpatient |
| 0211 | Coronary care-myocardial infraction | Inpatient |
| 0212 | Coronary care-pulmonary care | Inpatient |
| 0213 | Coronary care-heart transplant | Inpatient |
| 0214 | Coronary care-post CCU; redefined as intermediate CCU | Inpatient |
| 0219 | Coronary care-other coronary care | Inpatient |
| 0220 | Special charges-general classification | Unknown |
| 0221 | Special charges-admission charge | Unknown |
| 0222 | Special charges-technical support charge | Unknown |
| 0223 | Special charges-UR service charge | Unknown |
| 0224 | Special charges-late discharge, medically necessary | Unknown |
| 0229 | Special charges-other special charges | Unknown |
| 0230 | Incremental nursing charge rate-general classification | Inpatient |
| 0231 | Incremental nursing charge rate-nursery | Inpatient |
| 0232 | Incremental nursing charge rate-OB | Inpatient |
| 0233 | Incremental nursing charge rate-ICU (include transitional care) | Inpatient |
| 0234 | Incremental nursing charge rate-CCU (include transitional care) | Inpatient |
| 0235 | Incremental nursing charge rate-hospice | Other unclassified |
| 0239 | Incremental nursing charge rate-other | Inpatient |
| 0240 | All-inclusive ancillary-general classification | Unknown |
| 0241 | All-inclusive ancillary-basic | Unknown |
| 0242 | All-inclusive ancillary-comprehensive | Unknown |
| 0243 | All-inclusive ancillary-specialty | Unknown |
| 0249 | All-inclusive ancillary-other inclusive ancillary | Unknown |
| 0250 | Pharmacy-general classification | Other unclassified |
| 0251 | Pharmacy-generic drugs | Other unclassified |
| 0252 | Pharmacy-nongeneric drugs | Other unclassified |
| 0253 | Pharmacy-take home drugs | Other unclassified |
| 0254 | Pharmacy-drugs incident to other diagnostic service-subject | Other unclassified |
| 0255 | Pharmacy-drugs incident to radiology-subject to payment limit | Other unclassified |
| 0256 | Pharmacy-experimental drugs | Other unclassified |
| 0257 | Pharmacy-non-prescription | Other unclassified |
| 0258 | Pharmacy-IV solutions | Other unclassified |
| 0259 | Pharmacy-other pharmacy | Other unclassified |
| 0260 | IV therapy-general classification | Other unclassified |
| 0261 | IV therapy-infusion pump | Other unclassified |
| 0262 | IV therapy-pharmacy services | Other unclassified |
| 0263 | IV therapy-drug supply/delivery | Other unclassified |
| 0264 | IV therapy-supplies | Other unclassified |
| 0269 | IV therapy-other IV therapy | Other unclassified |
| 0270 | Medical/surgical supplies-general classification (also see 062X) | Other unclassified |
| 0271 | Medical/surgical supplies-nonsterile supply | Other unclassified |
| 0272 | Medical/surgical supplies-sterile supply | Other unclassified |
| 0273 | Medical/surgical supplies-take home supplies | Other unclassified |
| 0274 | Medical/surgical supplies-prosthetic/orthotic devices | Other unclassified |
| 0275 | Medical/surgical supplies-pace maker | Other unclassified |
| 0276 | Medical/surgical supplies-intraocular lens | Other unclassified |
| 0277 | Medical/surgical supplies-oxygen-take home | Other unclassified |
| 0278 | Medical/surgical supplies-other implants | Other unclassified |
| 0279 | Medical/surgical supplies-other devices | Other unclassified |
| 0280 | Oncology-general classification | Other unclassified |
| 0289 | Oncology-other oncology | Other unclassified |
| 0290 | DME (other than renal)-general classification | Other unclassified |
| 0291 | DME (other than renal)-rental | Other unclassified |
| 0292 | DME (other than renal)-purchase of new DME | Other unclassified |
| 0293 | DME (other than renal)-purchase of used DME | Other unclassified |
| 0294 | DME (other than renal)-related to and listed as DME | Other unclassified |
| 0299 | DME (other than renal)-other | Other unclassified |
| 0300 | Laboratory-general classification | Laboratory |
| 0301 | Laboratory-chemistry | Laboratory |
| 0302 | Laboratory-immunology | Laboratory |
| 0303 | Laboratory-renal patient (home) | Laboratory |
| 0304 | Laboratory-non-routine dialysis | Laboratory |
| 0305 | Laboratory-hematology | Laboratory |
| 0306 | Laboratory-bacteriology & microbiology | Laboratory |
| 0307 | Laboratory-urology | Laboratory |
| 0309 | Laboratory-other laboratory | Laboratory |
| 0310 | Laboratory pathological-general classification | Laboratory |
| 0311 | Laboratory pathological-cytology | Laboratory |
| 0312 | Laboratory pathological-histology | Laboratory |
| 0314 | Laboratory pathological-biopsy | Laboratory |
| 0319 | Laboratory pathological-other | Laboratory |
| 0320 | Radiology diagnostic-general classification | Laboratory |
| 0321 | Radiology diagnostic-angiocardiography | Laboratory |
| 0322 | Radiology diagnostic-arthrography | Laboratory |
| 0323 | Radiology diagnostic-arteriography | Laboratory |
| 0324 | Radiology diagnostic-chest X-ray | Laboratory |
| 0329 | Radiology diagnostic-other | Laboratory |
| 0330 | Radiology therapeutic-general classification | Laboratory |
| 0331 | Radiology therapeutic-chemotherapy injected | Laboratory |
| 0332 | Radiology therapeutic-chemotherapy oral | Laboratory |
| 0333 | Radiology therapeutic-radiation therapy | Laboratory |
| 0335 | Radiology therapeutic-chemotherapy IV | Laboratory |
| 0339 | Radiology therapeutic-other | Laboratory |
| 0340 | Nuclear medicine-general classification | Laboratory |
| 0341 | Nuclear medicine-diagnostic | Laboratory |
| 0342 | Nuclear medicine-therapeutic | Laboratory |
| 0349 | Nuclear medicine-other | Laboratory |
| 0350 | Computed tomographic (CT) scan-general classification | Laboratory |
| 0351 | CT scan-head scan | Laboratory |
| 0352 | CT scan-body scan | Laboratory |
| 0359 | CT scan-other CT scans | Laboratory |
| 0360 | Operating room services-general classification | Unknown |
| 0361 | Operating room services-minor surgery | Unknown |
| 0362 | Operating room services-organ transplant, other than kidney | Unknown |
| 0367 | Operating room services-kidney transplant | Unknown |
| 0369 | Operating room services-other operating room services | Unknown |
| 0370 | Anesthesia-general classification | Unknown |
| 0371 | Anesthesia-incident to RAD and subject to the payment limit | Unknown |
| 0372 | Anesthesia-incident to other diagnostic service and subject | Unknown |
| 0374 | Anesthesia-acupuncture | Unknown |
| 0379 | Anesthesia-other anesthesia | Unknown |
| 0380 | Blood-general classification | Laboratory |
| 0381 | Blood-packed red cells | Laboratory |
| 0382 | Blood-whole blood | Laboratory |
| 0383 | Blood-plasma | Laboratory |
| 0384 | Blood-platelets | Laboratory |
| 0385 | Blood-leukocytes | Laboratory |
| 0386 | Blood-other components | Laboratory |
| 0387 | Blood-other derivatives (cryoprecipitates) | Laboratory |
| 0389 | Blood-other blood | Laboratory |
| 0390 | Blood storage and processing-general classification | Laboratory |
| 0391 | Blood storage and processing-blood administration | Laboratory |
| 0399 | Blood storage and processing-other | Laboratory |
| 0400 | Other imaging services-general classification | Laboratory |
| 0401 | Other imaging services-diagnostic mammography | Laboratory |
| 0402 | Other imaging services-ultrasound | Laboratory |
| 0403 | Other imaging services-screening mammography | Laboratory |
| 0404 | Other imaging services-positron emission tomography | Laboratory |
| 0409 | Other imaging services-other | Laboratory |
| 0410 | Respiratory services-general classification | Other unclassified |
| 0412 | Respiratory services-inhalation services | Other unclassified |
| 0413 | Respiratory services-hyperbaric oxygen therapy | Other unclassified |
| 0419 | Respiratory services-other | Other unclassified |
| 0420 | Physical therapy-general classification | Other unclassified |
| 0421 | Physical therapy-visit charge | Other unclassified |
| 0422 | Physical therapy-hourly charge | Other unclassified |
| 0423 | Physical therapy-group rate | Other unclassified |
| 0424 | Physical therapy-evaluation or re-evaluation | Other unclassified |
| 0429 | Physical therapy-other | Other unclassified |
| 0430 | Occupational therapy-general classification | Other unclassified |
| 0431 | Occupational therapy-visit charge | Other unclassified |
| 0432 | Occupational therapy-hourly charge | Other unclassified |
| 0433 | Occupational therapy-group rate | Other unclassified |
| 0434 | Occupational therapy-evaluation or re-evaluation | Other unclassified |
| 0439 | Occupational therapy-other (may include restorative therapy) | Other unclassified |
| 0440 | Speech language pathology-general classification | Other unclassified |
| 0441 | Speech language pathology-visit charge | Other unclassified |
| 0442 | Speech language pathology-hourly charge | Other unclassified |
| 0443 | Speech language pathology-group rate | Other unclassified |
| 0444 | Speech language pathology-evaluation or re-evaluation | Other unclassified |
| 0449 | Speech language pathology-other | Other unclassified |
| 0450 | Emergency room - general classification | ED or UC |
| 0451 | Emergency room - EMTALA emergency medical screening services | ED or UC |
| 0452 | Emergency room - ER beyond EMTALA screening | ED or UC |
| 0456 | Emergency room-urgent care | ED or UC |
| 0459 | Emergency room-other | ED or UC |
| 0460 | Pulmonary function-general classification | Laboratory |
| 0469 | Pulmonary function-other | Laboratory |
| 0470 | Audiology-general classification | Laboratory |
| 0471 | Audiology-diagnostic | Laboratory |
| 0472 | Audiology-treatment | Laboratory |
| 0479 | Audiology-other | Laboratory |
| 0480 | Cardiology-general classification | Other unclassified |
| 0481 | Cardiology-cardiac cath lab | Other unclassified |
| 0482 | Cardiology-stress test | Other unclassified |
| 0483 | Cardiology-Echocardiology | Other unclassified |
| 0489 | Cardiology-other | Other unclassified |
| 0490 | Ambulatory surgical care-general classification | Outpatient |
| 0499 | Ambulatory surgical care-other | Outpatient |
| 0500 | Outpatient services-general classification | Outpatient |
| 0509 | Outpatient services-other | Outpatient |
| 0510 | Clinic-general classification | Outpatient |
| 0511 | Clinic-chronic pain center | Outpatient |
| 0512 | Clinic-dental center | Outpatient |
| 0513 | Clinic-psychiatric | Outpatient |
| 0514 | Clinic-OB-GYN | Outpatient |
| 0515 | Clinic-pediatric | Outpatient |
| 0516 | Clinic-urgent care clinic | ED or UC |
| 0517 | Clinic-family practice clinic | Outpatient |
| 0519 | Clinic-other | Outpatient |
| 0520 | Free-standing clinic-general classification | Outpatient |
| 0521 | Free-standing clinic-Clinic visit by a member to RHC/FQHC | Outpatient |
| 0522 | Free-standing clinic-Home visit by RHC/FQHC practitioner | Outpatient |
| 0523 | Free-standing clinic-family practice | Outpatient |
| 0524 | Free-standing clinic - visit by RHC/FQHC practitioner to a | Outpatient |
| 0525 | Free-standing clinic - visit by RHC/FQHC practitioner to a | Outpatient |
| 0526 | Free-standing clinic-urgent care (eff 10/96) | ED or UC |
| 0527 | Free-standing clinic-RHC/FQHC visiting nurse service(s) to a | Outpatient |
| 0528 | Free-standing clinic-visit by RHC/FQHC practitioner to other | Outpatient |
| 0529 | Free-standing clinic-other | Outpatient |
| 0530 | Osteopathic services-general classification | Outpatient |
| 0531 | Osteopathic services-osteopathic therapy | Outpatient |
| 0539 | Osteopathic services-other | Outpatient |
| 0540 | Ambulance-general classification | Other unclassified |
| 0541 | Ambulance-supplies | Other unclassified |
| 0542 | Ambulance-medical transport | Other unclassified |
| 0543 | Ambulance-heart mobile | Other unclassified |
| 0544 | Ambulance-oxygen | Other unclassified |
| 0545 | Ambulance-air ambulance | Other unclassified |
| 0546 | Ambulance-neo-natal ambulance | Other unclassified |
| 0547 | Ambulance-pharmacy | Other unclassified |
| 0548 | Ambulance-telephone transmission EKG | Other unclassified |
| 0549 | Ambulance-other | Other unclassified |
| 0550 | Skilled nursing-general classification | Unknown |
| 0551 | Skilled nursing-visit charge | Unknown |
| 0552 | Skilled nursing-hourly charge | Unknown |
| 0559 | Skilled nursing-other | Unknown |
| 0560 | Medical social services-general classification | Unknown |
| 0561 | Medical social services-visit charge | Unknown |
| 0562 | Medical social services-hourly charges | Unknown |
| 0569 | Medical social services-other | Unknown |
| 0570 | Home health aid (home health)-general classification | Other unclassified |
| 0571 | Home health aid (home health)-visit charge | Other unclassified |
| 0572 | Home health aid (home health)-hourly charge | Other unclassified |
| 0579 | Home health aid (home health)-other | Other unclassified |
| 0580 | Other visits (home health)-general classification (under | Other unclassified |
| 0581 | Other unclassified visits (home health)-visit charge (under HHPPS, not | Other unclassified |
| 0582 | Other visits (home health)-hourly charge (under HHPPS, not | Other unclassified |
| 0589 | Other visits (home health)-other (under HHPPS, not allowed as | Other unclassified |
| 0590 | Units of service (home health)-general classification (under | Other unclassified |
| 0599 | Units of service (home health)-other (under HHPPS, not allowed | Other unclassified |
| 0600 | Oxygen/Home Health-general classification | Other unclassified |
| 0601 | Oxygen/Home Health-stat or port equip/supply or count | Other unclassified |
| 0602 | Oxygen/Home Health-stat/equip/under 1 LPM | Other unclassified |
| 0603 | Oxygen/Home Health-stat/equip/over 4 LPM | Other unclassified |
| 0604 | Oxygen/Home Health-stat/equip/portable add-on | Other unclassified |
| 0610 | Magnetic resonance technology (MRT)-general classification | Laboratory |
| 0611 | MRT/MRI-brain (including brainstem) | Laboratory |
| 0612 | MRT/MRI-spinal cord (including spine) | Laboratory |
| 0614 | MRT/MRI-other | Laboratory |
| 0615 | MRT/MRA-Head and Neck | Laboratory |
| 0616 | MRT/MRA-Lower Extremities | Laboratory |
| 0618 | MRT/MRA-other | Laboratory |
| 0619 | MRT/Other MRI | Laboratory |
| 0621 | Medical/surgical supplies-incident to radiology-subject to the | Other unclassified |
| 0622 | Medical/surgical supplies-incident to other diagnostic service- | Other unclassified |
| 0623 | Medical/surgical supplies-surgical dressings - extension of 027X | Other unclassified |
| 0624 | Medical/surgical supplies-medical investigational devices and | Other unclassified |
| 0630 | Reserved | EXCLUDE |
| 0631 | Drugs requiring specific identification-single drug source | Other unclassified |
| 0632 | Drugs requiring specific identification-multiple drug source | Other unclassified |
| 0633 | Drugs requiring specific identification-restrictive prescription | Other unclassified |
| 0634 | Drugs requiring specific identification-EPO under 10,000 units | Other unclassified |
| 0635 | Drugs requiring specific identification-EPO 10,000 units or more | Other unclassified |
| 0636 | Drugs requiring specific identification-detailed coding | Other unclassified |
| 0637 | Self-administered drugs administered in an emergency situation - | Other unclassified |
| 0640 | Home IV therapy-general classification | Other unclassified |
| 0641 | Home IV therapy-nonroutine nursing | Other unclassified |
| 0642 | Home IV therapy-IV site care, central line | Other unclassified |
| 0643 | Home IV therapy-IV start/change peripheral line | Other unclassified |
| 0644 | Home IV therapy-nonroutine nursing, peripheral line | Other unclassified |
| 0645 | Home IV therapy-train patient/caregiver, central line | Other unclassified |
| 0646 | Home IV therapy-train disabled patient, central line | Other unclassified |
| 0647 | Home IV therapy-train patient/caregiver, peripheral line | Other unclassified |
| 0648 | Home IV therapy-train disabled patient, peripheral line | Other unclassified |
| 0649 | Home IV therapy-other IV therapy services | Other unclassified |
| 0650 | Hospice services-general classification | Other unclassified |
| 0651 | Hospice services-routine home care | Other unclassified |
| 0652 | Hospice services-continuous home care-1/2 | Other unclassified |
| 0655 | Hospice services-inpatient care | Other unclassified |
| 0656 | Hospice services-general inpatient care (non-respite) | Other unclassified |
| 0657 | Hospice services-physician services | Other unclassified |
| 0659 | Hospice services-other | Other unclassified |
| 0660 | Respite care (HHA)-general classification | Other unclassified |
| 0661 | Respite care (HHA)-hourly charge/skilled nursing | Other unclassified |
| 0662 | Respite care (HHA)-hourly charge/home health aide/homemaker | Other unclassified |
| 0670 | OP special residence charges - general classification | Outpatient |
| 0671 | OP special residence charges - hospital based | Outpatient |
| 0672 | OP special residence charges - contracted | Outpatient |
| 0679 | OP special residence charges - other special residence charges | Outpatient |
| 0700 | Cast room-general classification | Outpatient |
| 0709 | Cast room-other | Outpatient |
| 0710 | Recovery room-general classification | Unknown |
| 0719 | Recovery room-other | Unknown |
| 0720 | Labor room/delivery-general classification | Inpatient |
| 0721 | Labor room/delivery-labor | Inpatient |
| 0722 | Labor room/delivery-delivery | Inpatient |
| 0723 | Labor room/delivery-circumcision | Inpatient |
| 0724 | Labor room/delivery-birthing center | Inpatient |
| 0729 | Labor room/delivery-other | Inpatient |
| 0730 | EKG/ECG-general classification | Other unclassified |
| 0731 | EKG/ECG-Holter moniter | Other unclassified |
| 0732 | EKG/ECG-telemetry | Other unclassified |
| 0739 | EKG/ECG-other | Other unclassified |
| 0740 | EEG-general classification | Other unclassified |
| 0749 | EEG (electroencephalogram)-other | Other unclassified |
| 0750 | Gastro-intestinal services-general classification | Unknown |
| 0759 | Gastro-intestinal services-other | Unknown |
| 0760 | Treatment or observation room-general classification | Other unclassified |
| 0761 | Treatment or observation room-treatment room | Other unclassified |
| 0762 | Treatment or observation room-observation room | Other unclassified |
| 0769 | Treatment or observation room-other | Other unclassified |
| 0770 | Preventative care services-general classification | Unknown |
| 0771 | Preventative care services-vaccine administration | Unknown |
| 0779 | Preventative care services-other | Unknown |
| 0780 | Telemedicine - general classification | Outpatient |
| 0789 | Telemedicine - telemedicine | Outpatient |
| 0790 | Lithotripsy-general classification | Unknown |
| 0799 | Lithotripsy-other | Unknown |
| 0800 | Inpatient renal dialysis-general classification | Inpatient |
| 0801 | Inpatient renal dialysis-inpatient hemodialysis | Inpatient |
| 0802 | Inpatient renal dialysis-inpatient peritoneal (non-CAPD) | Inpatient |
| 0803 | Inpatient renal dialysis-inpatient CAPD | Inpatient |
| 0804 | Inpatient renal dialysis-inpatient CCPD | Inpatient |
| 0809 | Inpatient renal dialysis-other inpatient dialysis | Inpatient |
| 0810 | Organ acquisition-general classification | Unknown |
| 0811 | Organ acquisition-living donor | Unknown |
| 0812 | Organ acquisition-cadaver donor | Unknown |
| 0813 | Organ acquisition-unknown donor | Unknown |
| 0814 | Organ acquisition - unsuccessful organ search-donor bank charges | Unknown |
| 0815 | Allogeneic Stem Cell Acquisition/Donor Services | Unknown |
| 0819 | Organ acquisition-other donor | Unknown |
| 0820 | Hemodialysis OP or home dialysis-general classification | Outpatient |
| 0821 | Hemodialysis OP or home dialysis-hemodialysis-composite or other | Outpatient |
| 0822 | Hemodialysis OP or home dialysis-home supplies | Outpatient |
| 0823 | Hemodialysis OP or home dialysis-home equipment | Outpatient |
| 0824 | Hemodialysis OP or home dialysis-maintenance/100% | Outpatient |
| 0825 | Hemodialysis OP or home dialysis-support services | Outpatient |
| 0829 | Hemodialysis OP or home dialysis-other | Outpatient |
| 0830 | Peritoneal dialysis OP or home-general classification | Outpatient |
| 0831 | Peritoneal dialysis OP or home-peritoneal-composite or other rate | Outpatient |
| 0832 | Peritoneal dialysis OP or home-home supplies | Outpatient |
| 0833 | Peritoneal dialysis OP or home-home equipment | Outpatient |
| 0834 | Peritoneal dialysis OP or home-maintenance/100% | Outpatient |
| 0835 | Peritoneal dialysis OP or home-support services | Outpatient |
| 0839 | Peritoneal dialysis OP or home-other | Outpatient |
| 0840 | CAPD outpatient-general classification | Outpatient |
| 0841 | CAPD outpatient-CAPD/composite or other rate | Outpatient |
| 0842 | CAPD outpatient-home supplies | Outpatient |
| 0843 | CAPD outpatient-home equipment | Outpatient |
| 0844 | CAPD outpatient-maintenance/100% | Outpatient |
| 0845 | CAPD outpatient-support services | Outpatient |
| 0849 | CAPD outpatient-other | Outpatient |
| 0850 | CCPD outpatient-general classification | Outpatient |
| 0851 | CCPD outpatient-CCPD/composite or other rate | Outpatient |
| 0852 | CCPD outpatient-home supplies | Outpatient |
| 0853 | CCPD outpatient-home equipment | Outpatient |
| 0854 | CCPD outpatient-maintenance/100% | Outpatient |
| 0855 | CCPD outpatient-support services | Outpatient |
| 0859 | CCPD outpatient-other | Outpatient |
| 0880 | Miscellaneous dialysis-general classification | Unknown |
| 0881 | Miscellaneous dialysis-ultrafiltration | Unknown |
| 0882 | Miscellaneous dialysis-home dialysis aide visit | Outpatient |
| 0889 | Miscellaneous dialysis-other | Unknown |
| 0890 | Other donor bank-general classification; changed to reserved | EXCLUDE |
| 0891 | Other donor bank-bone; changed to reserved for national assignment | EXCLUDE |
| 0892 | Other donor bank-organ (other than kidney); changed to reserved for | EXCLUDE |
| 0893 | Other donor bank-skin; changed to reserved for national assignment | EXCLUDE |
| 0899 | Other donor bank-other; changed to reserved for national assignment | EXCLUDE |
| 0900 | Behavior Health Treatment/Services - general classification | Outpatient |
| 0901 | Behavior Health Treatment/Services - electroshock treatment | Outpatient |
| 0902 | Behavior Health Treatment/Services - milieu therapy (eff. 10/2004); | Outpatient |
| 0903 | Behavior Health Treatment/Services - play therapy (eff. 10/2004); | Outpatient |
| 0904 | Behavior Health Treatment/Services - activity therapy (eff. 10/2004); | Outpatient |
| 0905 | Behavior Health Treatment/Services - intensive outpatient services- | Outpatient |
| 0906 | Behavior Health Treatment/Services - intensive outpatient services- | Outpatient |
| 0907 | Behavior Health Treatment/Services - community behavioral health | Outpatient |
| 0909 | Reserved for National Use (eff. 10/2004); prior to 10/2004 defined | EXCLUDE |
| 0910 | Behavioral Health Treatment/Services-Reserved for National Assignment | EXCLUDE |
| 0911 | Behavioral Health Treatment/Services-rehabilitation (eff. 10/2004); | Inpatient |
| 0912 | Behavioral Health Treatment/Services-partial hospitalization-less | Inpatient |
| 0913 | Behavioral Health Treatment/Services-partial hospitalization- | Inpatient |
| 0914 | Behavioral Health Treatment/Services-individual therapy (eff. 10/2004) | Unknown |
| 0915 | Behavioral Health Treatment/Services-group therapy (eff. 10/2004); | Unknown |
| 0916 | Behavioral Health Treatment/Services-family therapy (eff. 10/2004); | Unknown |
| 0917 | Behavioral Health Treatment/Services-biofeedback (eff. 10/2004); | Unknown |
| 0918 | Behavioral Health Treatment/Services-testing (eff. 10/2004); prior | Unknown |
| 0919 | Behavioral Health Treatment/Services-other (eff. 10/2004); prior | Unknown |
| 0920 | Other diagnostic services-general classification | Laboratory |
| 0921 | Other diagnostic services-peripheral vascular lab | Laboratory |
| 0922 | Other diagnostic services-electromyelogram | Laboratory |
| 0923 | Other diagnostic services-pap smear | Laboratory |
| 0924 | Other diagnostic services-allergy test | Laboratory |
| 0925 | Other diagnostic services-pregnancy test | Laboratory |
| 0929 | Other diagnostic services-other | Laboratory |
| 0931 | Medical Rehabilitation Day Program - Half Day | Other unclassified |
| 0932 | Medical Rehabilitation Day Program - Full Day | Other unclassified |
| 0940 | Other therapeutic services-general classification | Other unclassified |
| 0941 | Other therapeutic services-recreational therapy | Other unclassified |
| 0942 | Other therapeutic services-education/training (include diabetes | Other unclassified |
| 0943 | Other therapeutic services-cardiac rehabilitation | Other unclassified |
| 0944 | Other therapeutic services-drug rehabilitation | Other unclassified |
| 0945 | Other therapeutic services-alcohol rehabilitation | Other unclassified |
| 0946 | Other therapeutic services-routine complex medical equipment | Other unclassified |
| 0947 | Other therapeutic services-ancillary complex medical equipment | Other unclassified |
| 0949 | Other therapeutic services-other | Other unclassified |
| 0951 | Professional Fees-athletic training (extension of 094X) | Unknown |
| 0952 | Professional Fees-kinesiotherapy (extension of 094X) | Unknown |
| 0960 | Professional fees-general classification | Unknown |
| 0961 | Professional fees-psychiatric | Outpatient |
| 0962 | Professional fees-ophthalmology | Unknown |
| 0963 | Professional fees-anesthesiologist (MD) | Unknown |
| 0964 | Professional fees-anesthetist (CRNA) | Unknown |
| 0969 | Professional fees-other (NOTE: 097X is an extension of 096X) | Unknown |
| 0971 | Professional fees-laboratory | Laboratory |
| 0972 | Professional fees-radiology diagnostic | Laboratory |
| 0973 | Professional fees-radiology therapeutic | Laboratory |
| 0974 | Professional fees-nuclear medicine | Laboratory |
| 0975 | Professional fees-operating room | Unknown |
| 0976 | Professional fees-respiratory therapy | Other unclassified |
| 0977 | Professional fees-physical therapy | Other unclassified |
| 0978 | Professional fees-occupational therapy | Other unclassified |
| 0979 | Professional fees-speech pathology (NOTE: 098X is an extension of | Other unclassified |
| 0981 | Professional fees-emergency room | ED or UC |
| 0982 | Professional fees-outpatient services | Outpatient |
| 0983 | Professional fees-clinic | Outpatient |
| 0984 | Professional fees-medical social services | Unknown |
| 0985 | Professional fees-EKG | Laboratory |
| 0986 | Professional fees-EEG | Laboratory |
| 0987 | Professional fees-hospital visit | Inpatient |
| 0988 | Professional fees-consultation | Unknown |
| 0989 | Professional fees-private duty nurse | Unknown |
| 0990 | Patient convenience items-general classification | Unknown |
| 0991 | Patient convenience items-cafeteria/guest tray | Unknown |
| 0992 | Patient convenience items-private linen service | Unknown |
| 0993 | Patient convenience items-telephone/telegraph | Unknown |
| 0994 | Patient convenience items-tv/radio | Unknown |
| 0995 | Patient convenience items-nonpatient room rentals | Unknown |
| 0996 | Patient convenience items-late discharge charge | Unknown |
| 0997 | Patient convenience items-admission kits | Unknown |
| 0998 | Patient convenience items-beauty shop/barber | Unknown |
| 0999 | Patient convenience items-other | Unknown |
| 1000 | Behavioral health Accommodations – general | Inpatient |
| 1001 | Behavioral health Accommodations – residential treatment psychiatric | Inpatient |
| 1002 | Behavioral health Accommodations – residential treatment chemical dependency | Inpatient |
| 2101 | Alternative Therapy Services – Acupuncture | Other unclassified |
| 2103 | Alternative Therapy Services – Massage | Other unclassified |
| 3101 | Adult Day Care – Medical and Social (hourly) | Other unclassified |
| 3103 | Adult Day Care – Medical and Social (daily) | Other unclassified |
| 3104 | Adult Day Care –Social (daily) | Other unclassified |
| 3109 | Adult Day Care –other | Other unclassified |
| 9000 | RUGS-no MDS assessment available | Post Acute or LTC |
| 9001 | Reduced physical functions-RUGS PA1/ADL index of 4-5 | Post Acute or LTC |
| 9002 | Reduced physical functions-RUGS PA2/ADL index of 4-5 | Post Acute or LTC |
| 9003 | Reduced physical functions-RUGS PB1/ADL index of 6-8 | Post Acute or LTC |
| 9004 | Reduced physical functions-RUGS PB2/ADL index of 6-8 | Post Acute or LTC |
| 9005 | Reduced physical functions-RUGS PC1/ADL index of 9-10 | Post Acute or LTC |
| 9006 | Reduced physical functions-RUGS PC2/ADL index of 9-10 | Post Acute or LTC |
| 9007 | Reduced physical functions-RUGS PD1/ADL index of 11-15 | Post Acute or LTC |
| 9008 | Reduced physical functions-RUGS PD2/ADL index of 11-15 | Post Acute or LTC |
| 9009 | Reduced physical functions-RUGS PE1/ADL index of 16-18 | Post Acute or LTC |
| 9010 | Reduced physical functions-RUGS PE2/ADL index of 16-18 | Post Acute or LTC |
| 9011 | Behavior only problems-RUGS BA1/ADL index of 4-5 | Post Acute or LTC |
| 9012 | Behavior only problems-RUGS BA2/ADL index of 4-5 | Post Acute or LTC |
| 9013 | Behavior only problems-RUGS BB1/ADL index of 6-10 | Post Acute or LTC |
| 9014 | Behavior only problems-RUGS BB2/ADL index of 6-10 | Post Acute or LTC |
| 9015 | Impaired cognition-RUGS IA1/ADL index of 4-5 | Post Acute or LTC |
| 9016 | Impaired cognition-RUGS IA2/ADL index of 4-5 | Post Acute or LTC |
| 9017 | Impaired cognition-RUGS IB1/ADL index of 6-10 | Post Acute or LTC |
| 9018 | Impaired cognition-RUGS IB2/ADL index of 6-10 | Post Acute or LTC |
| 9019 | Clinically complex-RUGS CA1/ADL index of 4-5 | Post Acute or LTC |
| 9020 | Clinically complex-RUGS CA2/ADL index of 4-5d | Post Acute or LTC |
| 9021 | Clinically complex-RUGS CB1/ADL index of 6-10 | Post Acute or LTC |
| 9022 | Clinically complex-RUGS CB2/ADL index of 6-10d | Post Acute or LTC |
| 9023 | Clinically complex-RUGS CC1/ADL index of 11-16 | Post Acute or LTC |
| 9024 | Clinically complex-RUGS CC2/ADL index of 11-16d | Post Acute or LTC |
| 9025 | Clinically complex-RUGS CD1/ADL index of 17-18 | Post Acute or LTC |
| 9026 | Clinically complex-RUGS CD2/ADL index of 17-18d | Post Acute or LTC |
| 9027 | Special care-RUGS SSA/ADL index of 7-13 | Post Acute or LTC |
| 9028 | Special care-RUGS SSB/ADL index of 14-16 | Post Acute or LTC |
| 9029 | Special care-RUGS SSC/ADL index of 17-18 | Post Acute or LTC |
| 9030 | Extensive services-RUGS SE1/1 procedure | Post Acute or LTC |
| 9031 | Extensive services-RUGS SE2/2 procedures | Post Acute or LTC |
| 9032 | Extensive services-RUGS SE3/3 procedures | Post Acute or LTC |
| 9033 | Low rehabilitation-RUGS RLA/ADL index of 4-11 | Post Acute or LTC |
| 9034 | Low rehabilitation-RUGS RLB/ADL index of 12-18 | Post Acute or LTC |
| 9035 | Medium rehabilitation-RUGS RMA/ADL index of 4-7 | Post Acute or LTC |
| 9036 | Medium rehabilitation-RUGS RMB/ADL index of 8-15 | Post Acute or LTC |
| 9037 | Medium rehabilitation-RUGS RMC/ADL index of 16-18 | Post Acute or LTC |
| 9038 | High rehabilitation-RUGS RHA/ADL index of 4-7 | Post Acute or LTC |
| 9039 | High rehabilitation-RUGS RHB/ADL index of 8-11 | Post Acute or LTC |
| 9040 | High rehabilitation-RUGS RHC/ADL index of 12-14 | Post Acute or LTC |
| 9041 | High rehabilitation-RUGS RHD/ADL index of 15-18 | Post Acute or LTC |
| 9042 | Very high rehabilitation-RUGS RVA/ADL index of 4-7 | Post Acute or LTC |
| 9043 | Very high rehabilitation-RUGS RVB/ADL index of 8-13 | Post Acute or LTC |
| 9044 | Very high rehabilitation-RUGS RVC/ADL index of 14-18 | Post Acute or LTC |
| 9019 | Clinically complex-RUGS CA1/ADL index of 11 | Post Acute or LTC |
| 9020 | Clinically complex-RUGS CA2/ADL index of 11D | Post Acute or LTC |
| 9021 | Clinically complex-RUGS CB1/ADL index of 12-16 | Post Acute or LTC |
| 9022 | Clinically complex-RUGS CB2/ADL index of 12-16D | Post Acute or LTC |
| 9023 | Clinically complex-RUGS CC1/ADL index of 17-18 | Post Acute or LTC |
| 9024 | Clinically complex-RUGS CC2/ADL index of 17-18D | Post Acute or LTC |
| 9025 | Special care-RUGS SSA/ADL index of 14 | Post Acute or LTC |
| 9026 | Special care-RUGS SSB/ADL index of 15-16 | Post Acute or LTC |
| 9027 | Special care-RUGS SSC/ADL index of 17-18 | Post Acute or LTC |
| 9028 | Extensive services-RUGS SE1/ADL index 7-18/1 procedure | Post Acute or LTC |
| 9029 | Extensive services-RUGS SE2/ADL index 7-18/2 procedures | Post Acute or LTC |
| 9030 | Extensive services-RUGS SE3/ADL index 7-18/3 procedures | Post Acute or LTC |
| 9031 | Low rehabilitation-RUGS RLA/ADL index of 4-13 | Post Acute or LTC |
| 9032 | Low rehabilitation-RUGS RLB/ADL index of 14-18 | Post Acute or LTC |
| 9033 | Low rehabilitation-RUGS RLA/ADL index of 4-11 | Post Acute or LTC |
| 9034 | Medium rehabilitation-RUGS RMB/ADL index of 8-14 | Post Acute or LTC |
| 9035 | Medium rehabilitation-RUGS RMC/ADL index of 15-18 | Post Acute or LTC |
| 9036 | High rehabilitation-RUGS RHA/ADL index of 4-7 | Post Acute or LTC |
| 9037 | High rehabilitation-RUGS RHB/ADL index of 8-12 | Post Acute or LTC |
| 9038 | High rehabilitation-RUGS RHC/ADL index of 13-18 | Post Acute or LTC |
| 9039 | Very High rehabilitation-RUGS RVA/ADL index of 4-8 | Post Acute or LTC |
| 9040 | Very high rehabilitation-RUGS RVB/ADL index of 9-15 | Post Acute or LTC |
| 9041 | Very high rehabilitation-RUGS RVC/ADL index of 16 | Post Acute or LTC |
| 9042 | Very high rehabilitation-RUGS RUA/ADL index of 4-8 | Post Acute or LTC |
| 9043 | Very high rehabilitation-RUGS RUB/ADL index of 9-15 | Post Acute or LTC |
| 9044 | Ultra high rehabilitation-RUGS RUC/ADL index of 16-18 | Post Acute or LTC |

Abbreviations: RCC – revenue center code; ED – emergency department; UC – urgent care; LTC – long term care facility.

Appendix S3. Place of service (POS) code to setting of diagnosis crosswalk.

| **POS** | **Place of Service Name** | **Broader Category** |
| --- | --- | --- |
| 01 | Pharmacy | Other unclassified |
| 02 | Telehealth Provided Other than in Patient’s Home | Outpatient |
| 03 | School | Other unclassified |
| 04 | Homeless Shelter | Other unclassified |
| 05 | Indian Health Service Free-standing Facility | Other unclassified |
| 06 | Indian Health Service Provider-based Facility | Other unclassified |
| 07 | Tribal 638 Free-standing Facility | Other unclassified |
| 08 | Tribal 638 Provider-based Facility | Other unclassified |
| 09 | Prison/Correctional Facility | Other unclassified |
| 10 | Telehealth Provided in Patient’s Home | Outpatient |
| 11 | Office | Outpatient |
| 12 | Home | Other unclassified |
| 13 | Assisted Living Facility | Post Acute or LTC |
| 14 | Group Home | Post Acute or LTC |
| 15 | Mobile Unit | Outpatient |
| 16 | Temporary Lodging | Other unclassified |
| 17 | Walk-in Retail Health Clinic | Outpatient |
| 18 | Place of Employment-Worksite | Outpatient |
| 19 | Off Campus-Outpatient Hospital | Outpatient |
| 20 | Urgent Care Facility | ED or UC |
| 21 | Inpatient Hospital | Inpatient |
| 22 | On Campus-Outpatient Hospital | Outpatient |
| 23 | Emergency Room – Hospital | ED or UC |
| 24 | Ambulatory Surgical Center | Outpatient |
| 25 | Birthing Center | Other unclassified |
| 26 | Military Treatment Facility | Other unclassified |
| 27-30 | Unassigned | Unknown |
| 31 | Skilled Nursing Facility | Post Acute or LTC |
| 32 | Nursing Facility | Post Acute or LTC |
| 33 | Custodial Care Facility | Post Acute or LTC |
| 34 | Hospice | Other unclassified |
| 35-40 | Unassigned | Unknown |
| 41 | Ambulance - Land | Other unclassified |
| 42 | Ambulance – Air or Water | Other unclassified |
| 43-48 | Unassigned | Unknown |
| 49 | Independent Clinic | Outpatient |
| 50 | Federally Qualified Health Center | Outpatient |
| 51 | Inpatient Psychiatric Facility | Inpatient |
| 52 | Psychiatric Facility-Partial Hospitalization | Outpatient |
| 53 | Community Mental Health Center | Outpatient |
| 54 | Intermediate Care Facility/ Individuals with Intellectual Disabilities | Post Acute or LTC |
| 55 | Residential Substance Abuse Treatment Facility | Inpatient |
| 56 | Psychiatric Residential Treatment Center | Inpatient |
| 57 | Non-residential Substance Abuse Treatment Facility | Outpatient |
| 58 | Non-residential Opioid Treatment Facility | Outpatient |
| 59 | Unassigned | Unknown |
| 60 | Mass Immunization Center | Other unclassified |
| 61 | Comprehensive Inpatient Rehabilitation Facility | Post Acute or LTC |
| 62 | Comprehensive Outpatient Rehabilitation Facility | Post Acute or LTC |
| 63-64 | Unassigned | Unknown |
| 65 | End-Stage Renal Disease Treatment Facility | Outpatient |
| 66-70 | Unassigned | Unknown |
| 71 | Public Health Clinic | Outpatient |
| 72 | Rural Health Clinic | Outpatient |
| 73-80 | Unassigned | Unknown |
| 81 | Independent Laboratory | Laboratory |
| 82-98 | Unassigned | Unknown |
| 99 | Other Place of Service | Other unclassified |

Abbreviations: ED – emergency department; UC – urgent care; LTC – long term care facility.

Appendix S4. National drug codes (NDC) of direct-acting antivirals.

| Proprietary Name | NDC |
| --- | --- |
| Epclusa | 61958-2201-1, 61958-2203-1 |
| Harvoni | 61958-1801-1, 61958-1803-1, 61958-1804-1, 61958-1805-1 |
| Ledipasvir and Sofosbuvir | 72626-2601-1 |
| Sofosbuvir and Velpatasvir | 72626-2701-1 |
| Sovaldi | 61958-1501-1, 61958-1503-1, 61958-1504-1, 61958-1505-1 |
| Vosevi | 61958-2401-1, 0074-2625-28, 0074-2625-56 |
| Mavyret | 0074-2625-01 |
| ZEPATIER | 0006-3074-02 |
| DAKLINZA | 0003-0011-01, 0003-0213-01, 0003-0215-01 |
| Viekira | 0074-0063-01, 0074-0063-28 |
| Viekira Pak | 0074-3093-01, 0074-3093-28 |
| Technivie | 0074-3082-28 |
| Olysio | 59676-225-07, 59676-225-28 |

Appendix S5. Assignment of setting of diagnosis – patients aged 18-64

104,454 HCV patients

55,999 with a single setting of diagnosis:

- 34,706 (62.0%) Lab
- 16,778 (30.0%) Outpatient
- 3,617 (6.5%) inpatient
- 486 (0.9%) Post-acute or LTC
- 209 (0.4%) ED or UC
- 176 (0.3%) other unclassified
- 27 （0.1） unknown

48,455 with multiple settings of diagnosis, after applying the hierarchical rule:

- 44,587 (92.0%) Outpatient
- 1,786 (3.7%) ED or UC
- 1,459 (3.0%) Other unclassified
- 326 (0.7%) inpatient
- 266 (0.6%) post acute or LTC
- 31 (0.1%) unknown

34,706 patients with lab as the initial setting of diagnosis

- 15,002 (43.2%) have additional HCV diagnoses within 30 days
  - 13,653 (39.3%) found a setting of diagnosis other than lab

Appendix S6. Assignment of setting of diagnosis – patients 65 or older

66,726 HCV patients

38,665 with a single setting of diagnosis:

- 24,139 (62.4%) Lab
- 12,129 (31.4%) Outpatient
- 1,680 (4.4%) Inpatient
- 485 (1.3%) Post-acute or LTC
- 164 (0.4%) Other unclassified
- 63 (0.2%) ED or UC
- 5 （0.0%） unknown

28,061 with multiple settings of diagnosis, after applying the hierarchical rule:

- 26,178 (93.3%) Outpatient
- 910 (3.2%) Other unclassified
- 634 (2.3%) ED or UC
- 200 (0.7%) Post acute or LTC
- 121 (0.4%) Inpatient
- 18 (0.1%) Unknown

24,139 patients with lab as the initial setting of diagnosis

- 10,049 (41.6%) have additional HCV diagnoses within 30 days
  - 9,000 (37.3%) found a setting of diagnosis other than lab

Appendix S7. Demographic, comorbidity, and DAA initiation characteristics of patients included in the study sample

|  | 18-64 | 65 or older |
| --- | --- | --- |
| N | 104,454 | 66,726 |
| Sex |  |  |
| Male | 60,021 (57.5%) | 37,242 (55.8%) |
| Female | 44,433 (42.5%) | 29,484 (44.2%) |
| Race/ethnicity |  |  |
| Non-Hispanic White | 69,101 (66.2%) | 38,320 (57.4%) |
| Non-Hispanic Black | 23,098 (22.1%) | 17,751 (26.6%) |
| Hispanic | 9,329 (8.9%) | 5,799 (8.7%) |
| Asian/Pacific Islander | 817 (0.8%) | 3,112 (4.7%) |
| Other or Unknown | 2,109 (2.0%) | 1,744 (2.6%) |
| Age |  |  |
| >=18 and <35 | 5,167 (5.0%) |  |
| >=35 and <55 | 39,786 (38.1%) |  |
| >=55 and <65 | 59,501 (57.0%) |  |
| >=65 and <75 |  | 54,751 (82.1%) |
| >=75 |  | 11,975 (18.0%) |
| Medicare-Medicaid dual enrollment |  |  |
| Yes | 84,886 (81.3%) | 33,211 (49.8%) |
| No | 19,568 (18.7%) | 33,515 (50.2%) |
| Comorbidity |  |  |
| Opioid Use Disorder | 24,704 (23.7%) | 5,418 (8.1%) |
| Alcohol Use Disorder | 20,728 (19.8%) | 6,215 (9.3%) |
| Advance Liver Disease | 17,530 (16.8%) | 12,971 (19.4%) |
| HIV | 8,023 (7.7%) | 2,038 (3.1%) |
| Mental Health Disorder | 71,353 (68.3%) | 27,659 (41.5%) |
| DAA Initiation within 180 Days | 31,370 (30.0%) | 21,332 (32.0%) |

Note: HIV - human immunodeficiency virus; DAA – direct-acting antiviral.

Appendix S8 Association between setting of diagnosis and HCV treatment initiation within 180 days adjusting for covariates in logistic regression.

| Patients | Setting of diagnosis | Marginal Effect | 95% CI | p-value |
| --- | --- | --- | --- | --- |
| Age 18-64 | Outpatient | Ref | | |
|  | Emergency department/urgent care | -0.216 | (-0.230, -0.201) | <0.001 |
|  | Hospital | -0.264 | (-0.273, -0.255) | <0.001 |
|  | Laboratory or unknown | -0.151 | (-0.157, -0.144) | <0.001 |
|  | Other unclassified | -0.146 | (-0.161, -0.132) | <0.001 |
| Age >=65 | Outpatient | Ref | | |
|  | Emergency department/urgent care | -0.242 | (-0.267, -0.218) | <0.001 |
|  | Hospital | -0.292 | (-0.305, -0.278) | <0.001 |
|  | Laboratory or unknown | -0.196 | (-0.204, -0.189) | <0.001 |
|  | Other unclassified | -0.186 | (-0.203,-0.168) | <0.001 |

Note: Marginal effects were generated using multivariate logistic regressions with the algorithm-based setting of diagnosis as the independent variable and treatment initiation as the dependent variable. Other covariates adjusted for in the model included the year of diagnosis, patient demographics, and comorbidities.

Appendix S9 Association between setting of diagnosis and HCV treatment initiation within 90 days adjusting for covariates in logistic regression.

| Patients | Setting of diagnosis | Marginal Effect | 95% CI | p-value |
| --- | --- | --- | --- | --- |
| Age 18-64 | Outpatient | Ref | | |
|  | Emergency department/urgent care | -0.169 | (-0.180, -0.157) | <0.001 |
|  | Hospital | -0.203 | (-0.210, -0.196) | <0.001 |
|  | Laboratory or unknown | -0.122 | (-0.128, -0.117) | <0.001 |
|  | Other unclassified | -0.114 | (-0.127, -0.102) | <0.001 |
| Age >=65 | Outpatient | Ref | | |
|  | Emergency department/urgent care | -0.191 | (-0.211, -0.170) | <0.001 |
|  | Hospital | -0.231 | (-0.242, -0.221) | <0.001 |
|  | Laboratory or unknown | -0.160 | (-0.166, -0.153) | <0.001 |
|  | Other unclassified | -0.149 | (-0.164, -0.134) | <0.001 |

Note: Marginal effects were generated using multivariate logistic regressions with the algorithm-based setting of diagnosis as the independent variable and treatment initiation as the dependent variable. Other covariates adjusted for in the model included the year of diagnosis, patient demographics, and comorbidities.

Appendix S10 Association between setting of diagnosis and HCV treatment initiation within 270 days adjusting for covariates in logistic regression.

| Patients | Setting of diagnosis | Marginal Effect | 95% CI | p-value |
| --- | --- | --- | --- | --- |
| Age 18-64 | Outpatient | Ref | | |
|  | Emergency department/urgent care | -0.227 | (-0.242, -0.211) | <0.001 |
|  | Hospital | -0.280 | (-0.290, -0.269) | <0.001 |
|  | Laboratory or unknown | -0.158 | (-0.164, -0.151) | <0.001 |
|  | Other unclassified | -0.150 | (-0.165, -0.134) | <0.001 |
| Age >=65 | Outpatient | Ref | | |
|  | Emergency department/urgent care | -0.259 | (-0.285, -0.234) | <0.001 |
|  | Hospital | -0.300 | (-0.315, -0.285) | <0.001 |
|  | Laboratory or unknown | -0.208 | (-0.216, -0.200) | <0.001 |
|  | Other unclassified | -0.199 | (-0.217, -0.181) | <0.001 |

Note: Marginal effects were generated using multivariate logistic regressions with the algorithm-based setting of diagnosis as the independent variable and treatment initiation as the dependent variable. Other covariates adjusted for in the model included the year of diagnosis, patient demographics, and comorbidities.

Appendix S11 Demographic, comorbidity, and DAA initiation characteristics of patients by the algorithm-based setting of diagnosis, using an alternative definition for HCV diagnosis

|  | ED or UC | Hospital | Outpatient | Other Unclassified | Laboratory or Unknown | p-value a |
| --- | --- | --- | --- | --- | --- | --- |
| a. Aged 18-64 at diagnosis |  |  |  |  |  |  |
| N | 1,869 | 4,430 | 59,865 | 2,436 | 11,817 |  |
| Year of diagnosis |  |  |  |  |  | <0.001 |
| 2015 | 1.9% | 4.3% | 75.1% | 2.8% | 15.9% |  |
| 2016 | 2.3% | 5.5% | 74.2% | 3.1% | 14.9% |  |
| 2017 | 2.6% | 6.2% | 73.9% | 3.1% | 14.4% |  |
| 2018 | 2.9% | 7.3% | 73.9% | 3.4% | 12.5% |  |
| 2019 | 2.6% | 7.5% | 74.7% | 2.9% | 12.3% |  |
| Sex |  |  |  |  |  | <0.001 |
| Male | 53.7% | 59.6% | 58.5% | 59.6% | 58.3% |  |
| Female | 46.3% | 40.4% | 41.5% | 40.4% | 41.7% |  |
| Race |  |  |  |  |  | <0.001 |
| White | 64.1% | 59.4% | 66.1% | 64.9% | 65.8% |  |
| Black | 23.8% | 26.1% | 22.7% | 24.9% | 22.4% |  |
| Hispanic | 9.3% | 11.0% | 8.5% | 7.6% | 9.5% |  |
| Asian/Pacific islander | 0.4% | 0.9% | 0.7% | 1.1% | 0.7% |  |
| Other or unknown | 2.4% | 2.5% | 2.0 % | 1.5% | 1.6% |  |
| Age |  |  |  |  |  | <0.001 |
| >=18 and <35 | 8.3% | 9.0% | 4.6% | 2.6% | 4.7% |  |
| >=35 and <55 | 43.2% | 39.6% | 37.5% | 31.2% | 37.3% |  |
| >=55 and <65 | 48.5% | 51.4% | 58.0% | 66.2% | 58.0% |  |
| >=65 and <75 |  |  |  |  |  |  |
| >=75 |  |  |  |  |  |  |
| Medicare-Medicaid dual enrollment |  |  |  |  |  | <0.001 |
| Yes | 86.4% | 85.5% | 80.9% | 84.8% | 81.1% |  |
| No | 13.6% | 14.5% | 19.1% | 15.2% | 18.9% |  |
| Comorbidity |  |  |  |  |  |  |
| Opioid use disorder | 37.0% | 39.6% | 22.6% | 21.3% | 22.7% | <0.001 |
| Alcohol use disorder | 34.0% | 36.9% | 19.8% | 23.5% | 17.8 % | <0.001 |
| Advance liver disease | 24.5% | 25.7% | 17.6% | 30.3% | 16.5% | <0.001 |
| HIV | 9.8% | 10.0% | 7.4% | 6.9% | 8.2% | <0.001 |
| Mental health disorder | 81.7% | 79.9% | 67.3% | 77.5% | 65.8% | <0.001 |
| DAA initiation within 180 days | 15.6% | 8.4% | 42.3% | 26.4% | 33.7% | <0.001 |
|  |  |  |  |  |  |  |
| b. 65 or older at diagnosis |  |  |  |  |  |  |
| N | 656 | 2,040 | 37,516 | 1,681 | 7,356 |  |
| Year of diagnosis |  |  |  |  |  | <0.001 |
| 2015 | 1.0% | 2.8% | 76.7% | 2.8% | 16.8% |  |
| 2016 | 1.3% | 4.0% | 75.9% | 3.1% | 15.7% |  |
| 2017 | 1.3% | 4.2% | 76.8% | 3.6% | 14.0% |  |
| 2018 | 1.9% | 5.6% | 75.3% | 3.9% | 13.3% |  |
| 2019 | 1.3% | 5.5% | 75.7% | 4.8% | 12.7% |  |
| Sex |  |  |  |  |  | <0.001 |
| Male | 59.6% | 38.3% | 56.6% | 61.5% | 55.2% |  |
| Female | 40.4% | 61.7% | 43.4% | 38.6% | 44.8% |  |
| Race |  |  |  |  |  | <0.001 |
| White | 48.9% | 49.6% | 56.1% | 53.9% | 57.5% |  |
| Black | 37.8% | 35.3% | 28.4% | 32.7% | 25.8% |  |
| Hispanic | 8.7% | 9.7% | 8.7% | 8.8% | 8.8% |  |
| Asian/Pacific islander | 2.1% | 3.0% | 4.4% | 2.7% | 5.3% |  |
| Other or unknown | 2.4% | 2.5% | 2.5% | 1.9% | 2.6% |  |
| Age |  |  |  |  |  | <0.001 |
| >=18 and <35 |  |  |  |  |  |  |
| >=35 and <55 |  |  |  |  |  |  |
| >=55 and <65 |  |  |  |  |  |  |
| >=65 and <75 | 83.8% | 81.3% | 82.4% | 79.7% | 81.5% |  |
| >=75 | 16.2% | 18.7% | 17.7% | 20.4% | 18.5% |  |
| Dual eligibility |  |  |  |  |  | <0.001 |
| Yes | 68.3% | 62.7% | 50.3% | 70.8% | 50.9% |  |
| No | 31.7% | 37.3% | 49.7% | 29.2% | 49.1% |  |
| Comorbidity |  |  |  |  |  |  |
| Opioid use disorder | 19.7% | 17.8% | 7.8% | 11.0% | 7.9% | <0.001 |
| Alcohol use disorder | 22.0% | 20.5% | 9.4% | 16.7% | 8.2% | <0.001 |
| Advance liver disease | 30.2% | 32.3% | 20.6% | 29.3% | 20.4% | <0.001 |
| HIV | 3.8% | 5.8% | 2.9% | 4.6% | 2.8% | <0.001 |
| Mental health disorder | 60.2% | 62.5% | 40.1% | 65.7% | 40.2% | <0.001 |
| DAA initiation within 6 months | 16.3% | 8.8% | 46.8% | 24.2% | 36.0% | <0.001 |

Note: This table shows the sample size, demographic covariates and comorbidities for new HCV patients diagnosed in different settings. HCV diagnosis was defined as an RNA test followed by 2 ICD diagnoses within 180 days and on different service dates. Separate tables were reported for patients aged 18-64 and 65 or older. RTI definition was used for the race-ethnicity variable. P values were calculated using Chi-squared test.

Abbreviations. ED, emergency department. UC, urgent care clinic. DAA, direct-acting antiviral. HIV, human immunodeficiency virus.

ap-value for chi-square test.

Appendix S12 Association between setting of diagnosis and HCV treatment initiation within 180 days adjusting for covariates in logistic regression, using an alternative definition for HCV diagnosis.

| Patients | Setting of diagnosis | Marginal Effect | 95% CI | p-value |
| --- | --- | --- | --- | --- |
| Age 18-64 | Outpatient | Ref | | |
|  | Emergency department/urgent care | -0.251 | (-0.269, -0.233) | <0.001 |
|  | Hospital | -0.329 | (-0.339, -0.319) | <0.001 |
|  | Laboratory or unknown | -0.085 | (-0.094, -0.075) | <0.001 |
|  | Other unclassified | -0.161 | (-0.178, -0.143) | <0.001 |
| Age >=65 | Outpatient | Ref | | |
|  | Emergency department/urgent care | -0.287 | (-0.318, -0.256) | <0.001 |
|  | Hospital | -0.368 | (-0.382, -0.354) | <0.001 |
|  | Laboratory or unknown | -0.107 | (-0.119, -0.095) | <0.001 |
|  | Other unclassified | -0.203 | (-0.225, -0.180) | <0.001 |

Note: Marginal effects were generated using multivariate logistic regressions with the algorithm-based setting of diagnosis as the independent variable and treatment initiation as the dependent variable. Other covariates adjusted for in the model included year of diagnosis, patient demographics, and comorbidities. HCV diagnosis was defined as an RNA test followed by 2 ICD diagnoses within 180 days and on different service dates.
